# Recent Advances in Aptamer-Based Biosensors for Detection of *Pseudomonas aeruginosa*

**DOI:** 10.3389/fmicb.2020.605229

**Published:** 2020-12-22

**Authors:** Xin Zheng, Shunxiang Gao, Jihong Wu, Xiaobo Hu

**Affiliations:** ^1^Department of Clinical Laboratory, Longhua Hospital, Shanghai University of Traditional Chinese Medicine, Shanghai, China; ^2^Department of Ophthalmology, Eye Institute, Eye and ENT Hospital, Fudan University, Shanghai, China; ^3^State Key Laboratory of Medical Neurobiology, MOE Frontiers Center for Brain Science, Institute of Brain Science, Fudan University, Shanghai, China; ^4^NHC Key Laboratory of Myopia (Fudan University), Key Laboratory of Myopia, Chinese Academy of Medical Sciences, Shanghai Key Laboratory of Visual Impairment and Restoration, Shanghai, China

**Keywords:** *Pseudomonas aeruginosa*, aptamer, molecular recognition, biosensor, detection

## Abstract

Increasing concerns about nosocomial infection, food and environmental safety have prompted the development of rapid, accurate, specific and ultrasensitive methods for the early detection of critical pathogens. *Pseudomonas aeruginosa* is one of the most common pathogens that cause infection. It is ubiquitous in nature, being found in water, soil, and food, and poses a great threat to public health. The conventional detection technologies are either time consuming or readily produce false positive/negative results, which makes them unsuitable for early diagnosis and spot detection of *P. aeruginosa*. To circumvent these drawbacks, many efforts have been made to develop biosensors using aptamers as bio-recognition elements. Various aptamer-based biosensors for clinical diagnostics, food, and environmental monitoring of *P. aeruginosa* have been developed in recent years. In this review, we focus on the latest advances in aptamer-based biosensors for detection of *P. aeruginosa*. Representative biosensors are outlined according to their sensing mechanisms, which include optical, electrochemical and other signal transduction methods. Possible future trends in aptamer biosensors for pathogen detection are also outlined.

## Introduction

*Pseudomonas aeruginosa*, an opportunistic Gram-negative bacterium, is one of the most intractable multidrug-resistant bacteria causing severe nosocomial infections and poses an increasing threat to human health (Curran et al., 2018; Horcajada et al., 2019; Jean et al., 2020). In 2017, *P. aeruginosa* was listed as a critical pathogen by the World Health Organization. It is widely distributed in soil, water, air, animals and humans. Its widespread habitats make it difficult to control. Immunocompromised patients or people with damaged tissue barriers are often susceptible to serious *P. aeruginosa* infection, which causes diseases such as keratitis, endophthalmitis, pneumonia, urinary tract infections, lung diseases, cystic fibrosis, neutropenia, and sepsis (Winstanley et al., 2016; Paz-Zarza et al., 2019; Tummler, 2019; Kowalski et al., 2020). Moreover, *P. aeruginosa* tends to form biofilm, which is difficult to eradicate and has 10- to 1,000-fold higher resistance to antibiotic concentrations compared with equivalent floating bacteria (Lewis, 2001; Pang et al., 2019). Therefore, rapid, accurate, and ultrasensitive identification of *P. aeruginosa* is critical for both empirical antipseudomonal therapy and the guarantee of food security.

Diagnostic methods for *P. aeruginosa* presently include conventional culture-dependent colony counting, immunodetection, polymerase chain reaction (PCR), and matrix-assisted laser desorption/ionization time-of-flight mass spectrometry (MALDI-TOF-MS) (Rajapaksha et al., 2019). The traditional culture-based approach, regarded as the golden standard for pathogenic microorganism detection, is the most commonly used method (Perry, 2017). However, it is labor-intensive and time consuming (48–120 h), which makes it unsuitable for rapid detection of *P. aeruginosa* (Varadi et al., 2017). Immunological tests are also commonly used for clinical diagnoses. However, they are dependent on antibodies, which are sensitive to temperature and pH changes, and prone to false negatives/positives (Mauch and Levy, 2014). Another approach for *P. aeruginosa* detection is PCR-based method, which requires strict protocols (Deschaght et al., 2011). MALDI-TOF-MS requires sophisticated instrumentation and highly trained personnel. Bacterial enrichment is also needed before analysis, resulting in a long turnaround time (Perry, 2017). Therefore, the development of accurate, sensitive, specific, and rapid methods for identifying *P. aeruginosa* remains a challenging research goal. Biosensors are promising alternatives for pathogen detection (Khater et al., 2017; Nasseri et al., 2018; Silva et al., 2018; Furst and Francis, 2019; Cesewski and Johnson, 2020). In recent years, biosensors have been widely used in the microbial field, because of their rapidity, sensitivity, high specificity, and low cost (Farooq et al., 2018; Zhang et al., 2019a). In general, specific interactions between molecular recognition elements and pathogens can be converted into electrical, optical, or other signal outputs for analysis and detection. Therefore, the bio-recognition element is the most critical part in biosensors (Morales and Halpern, 2018; Zhang and Tian, 2018). Antibodies have been used as recognition elements in biosensors for decades. However, antibody development requires a tedious and complicated *in vivo* screening process, which leads to long development time and high cost. Moreover, antibodies are sensitive to temperature and pH changes and prone to denaturation (Alahi and Mukhopadhyay, 2017). Thus, researchers have sought to identify new molecular recognition elements that can retain the high affinity and specificity of antibodies without their shortcomings.

Aptamer, a promising alternative to antibodies as molecular recognition tools, has spurred a great interest since 1990 (Ellington and Szostak, 1990; Tuerk and Gold, 1990; Tan et al., 2020). They are short single-stranded DNA or RNA, selected *in vitro* from a synthesized random library using systematic evolution of ligands by exponential enrichment (SELEX) (Wang C. H. et al., 2019a; Wang T. et al., 2019; Yan et al., 2019). By folding into specific tertiary structures, aptamers bind to their targets with high affinity and specificity by complementary shape interactions (Gao et al., 2019). Compared with antibodies, aptamer-based biosensors have many advantages, including lower cost, less batch-to-batch variation, automated chemical synthesis, and more flexible modification strategies (Radom et al., 2013; Groff et al., 2015; Moutsiopoulou et al., 2019; Chen et al., 2020). Furthermore, targets such as toxins or haptens cannot be used to produce antibodies with high affinity, and in these cases aptamers possess a significant advantage. These excellent characteristics provide an opportunity for aptamers to replace antibodies in all analytical formats. To date, a myriad of aptamer-based biosensors have been developed to detect various bacteria, such as *Escherichia coli* (Hua et al., 2018; Dong et al., 2020; Kaur et al., 2020; Wang et al., 2020), *Salmonella enterica* (Fang et al., 2014; Chinnappan et al., 2017; Dao et al., 2018; Luo et al., 2020), and *Staphylococcus aureus* (Shrivastava et al., 2018; Cai et al., 2020; Fu et al., 2020; Pla et al., 2020). For *P. aeruginosa*, multiple aptamer-based sensors (aptasensors) have also been developed using optical, electrochemical and other signal sensing approaches. In this review, we first briefly introduce aptamers that have been selected against *P. aeruginosa*. Subsequently, we focus on the latest advances in aptamer-based biosensors detection of *P. aeruginosa*. Finally, the current challenges and future trends of aptamer-based biosensors in *P. aeruginosa* detection are further discussed.

## *P. aeruginosa* Aptamers Selected in Recent Years

**FIGURE 1:**
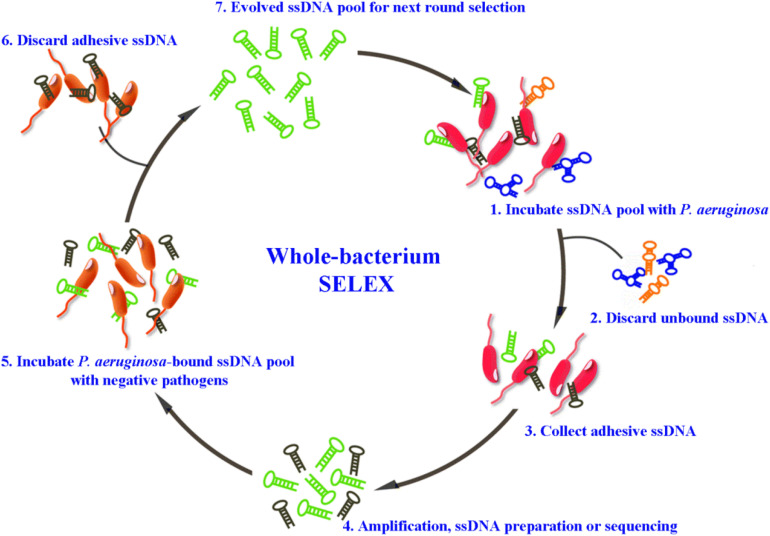
Schematic representation of whole-bacterium aptamer screening in *P. aeruginosa* using systematic evolution of ligands by exponential enrichment (SELEX) (Liu et al., 2020).

For the detection of *P. aeruginosa*, several specific aptamers have so far been isolated. Wang et al. (2011) selected an ssDNA aptamer specifically binding to *P. aeruginosa* (ATCC 27853) using a whole-bacterium SELEX strategy. Aptamers obtained through such kind of derived SELEX strategy (for example Figure 1), can bind to targets on bacteria in their native and most stable conformation, without changes before selection (Liu et al., 2020). The most specific aptamer, named F23, can identify the target bacteria with a low nanomolar dissociation constant (*K*_d_ = 17.27 ± 5.00 nM). F23 laid the foundation for many sensitive and rapid detection methods for *P. aeruginosa*. Similarly, another specific aptamer against *P. aeruginosa* (ATCC 27853) with a *K*_d_ of 15.16 ± 3.62 nM was obtained by other researchers (Wang et al., 2018). Moreover, Soundy and Day (2017) obtained three chimeric DNA aptamers, with *K*_d_ values in the nanomolar range, against live biofilm-derived PA692 *P. aeruginosa* cells. Although these aptamers were derived from biofilms, they can bind to both planktonic- and biofilm-grown cells. Another interesting example is provided by Davydova et al. (2017) who developed a 2′-fluoro RNA aptamer capable of internalizing into *P. aeruginosa* cells. As specific bio-recognition molecules, these above aptamers have provided effective tools for the rapid detection, early diagnosis, and therapeutic targeting of *P. aeruginosa.*

## Aptasensors Based on Optical Transduction

Optical methods have been widely applied in various fields, owing to their convenience, low-cost, rapidity, and resistance to electrical noise. They can be divided into several subclasses, which are based on absorption, reflection, refraction, dispersion, infrared, chemiluminescence, and fluorescence (Paniel et al., 2013). Among them, fluorescence spectroscopy, surface plasmon resonance (SPR), and surface-enhanced Raman scattering (SERS) are the most employed optical techniques because of their enhanced sensitivity.

### Fluorescence-Based Aptasensors

After an electron is promoted to an excited state by absorbing a high-energy photon, fluorescence emission of a lower-energy photon occurs when the electron returns to its original ground state (Chen et al., 2018). Aptamer-based fluorescent biosensors mainly detect their targets through changes in fluorescence polarization or intensity that result from the interaction between the fluorescently labeled aptamer and its corresponding target.

**FIGURE 2:**
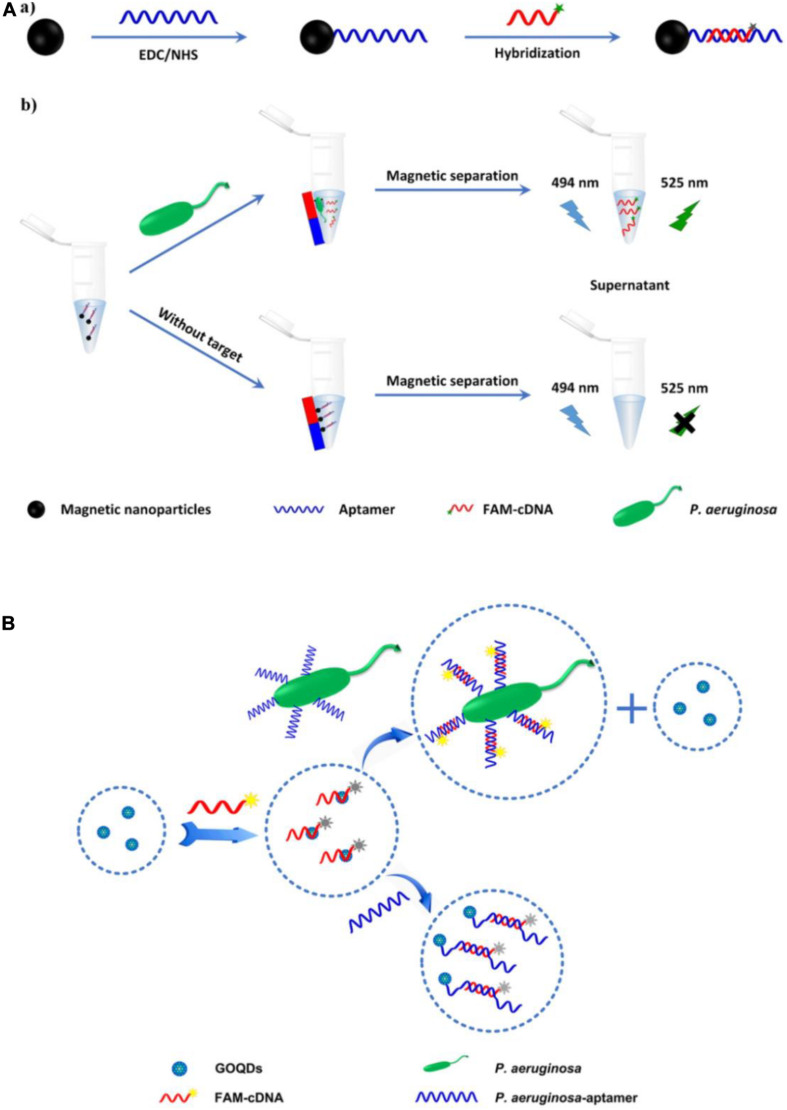
**(A)** Schematic illustration of the fabrication of magnetic nanoparticles modified by the hybrid of aptamer and carboxyfluorescein-labeled complementary DNA **(a)**, and the principle of *P. aeruginosa* detection **(b)** (Zhong et al., 2018). **(B)** Schematic illustration of the principle of fluorescent aptasensor detection of *P. aeruginosa* (Gao et al., 2018).

Traditional organic fluorescent dyes, which have long been used in sensors, include carboxyfluorescein (FAM), fluorescein isothiocyanate (FITC), and acridine orange (Paniel et al., 2013). A fluorescence biosensor based on an FITC-aptamer was developed by Kim et al. (2013) for *P*. *aeruginosa* detection. The aptasensor has been used to analyze drinking water, with a lower limit of detection (LOD) of 5.07 colony-forming units (CFU)/mL. However, its linear range (5.64 to 10^2^ CFU/mL) is suboptimal. Another fluorescence aptasensor was reported, based on aptamer hybridization with carboxyfluorescein-labeled complementary DNA (FAM-cDNA) in combination with magnetic separation (Figure 2A) (Zhong et al., 2018). In the absence of *P. aeruginosa*, FAM-cDNA hybridizes with the aptamer, which is covalently bound to the surface of magnetic nanoparticles (MNPs). In the presence of *P. aeruginosa*, FAM-cDNA will be displaced from the aptamer by the bacterium and therefore released from the MNPs. The quantification of *P. aeruginosa* can then be determined from the fluorescence intensity of the FAM-cDNA in the supernatant after magnetic separation. The assay has an outstanding linear response to the logarithm of *P. aeruginosa* concentrations ranging from 10 to 10^8^ CFU/mL, with a LOD as low as 1 CFU/mL. The whole detection process can be finished within 1.5 h. Gao et al. (2018) likewise engineered a fluorescent aptasensor based on the same DNA hybridization principle, with graphene oxide quantum dots (GOQDs) serving as the quencher. As shown in Figure 2B, upon addition of *P. aeruginosa*, the aptamer specifically binds to it as a bio-recognition element. FAM-cDNA prefers to hybridize with the aptamer, resulting in the desorption of FAM-cDNA from GOQDs and recovery of FAM fluorescence. The aptasensor shows a linear response to *P. aeruginosa* concentration in the range from 1.28 × 10^3^ to 2.00 × 10^7^ CFU/mL, with a LOD of 100 CFU/mL. The system can complete all detection steps within 2 h and has been used to detect *P. aeruginosa* in drinking water, orange juice, and popsicle samples. Although organic fluorescent dyes are still the most widely used fluorescent dyes in biosensors currently, there exist some well-known disadvantages, such as interference from background fluorescence, short lifetime as well as poor stability against photo-bleaching. It is crucial to solve these problems for their further application in rapid detection of pathogens.

**FIGURE 3:**
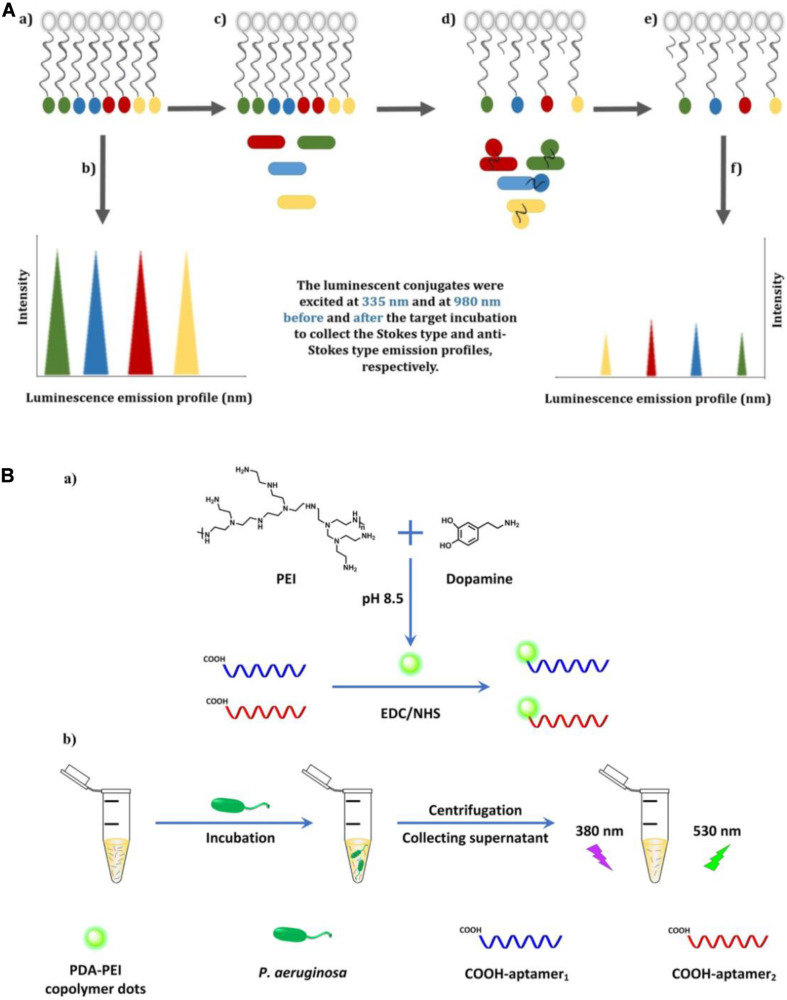
**(A)** Illustration of the multiplex-detection of conjugates of luminescent upconversion nanoparticles and quantum dots **(a)**, luminescence emission profiles of the conjugates before interaction with the targets **(b)**, addition of the targets into the conjugate solution **(c)**, binding of the luminescent aptamer–nanoparticle probes to the targets **(d)**, separation of the unbound conjugates **(e)**, and luminescence emission profiles of the conjugates after interaction with the targets **(f)** (Yüce et al., 2018). **(B)** Schematic illustration of the fabrication of dual-aptamer-labeled polydopamine–polyethyleneimine (PDA-PEI) probe **(a)**, and the detection procedure for *P*. *aeruginosa* **(b)** (Zhong et al., 2020).

Compared with organic dyes, fluorescent nanomaterials possess numerous advantageous characteristics, such as a broader absorption spectrum, a narrower and size-tunable emission spectrum, and better stability against photobleaching (Yao et al., 2014). Owing to their attractive properties, fluorescent nanomaterials, such as carbon dots (CDs), quantum dots (QDs), and upconversion nanoparticles (UCNPs), have been widely applied in biosensor fabrication (Chen et al., 2018). Wang et al. (2018) constructed an aptamer-based fluorescence assay, in which the specific aptamer was conjugated with photoluminescent CDs as the fluorescent probe and graphene oxide (GO) served as the anchor and quencher. The use of GO can reduce the background signals of CDs and improve sensitivity. This aptasensor displayed excellent specificity toward *P. aeruginosa*, with a lower LOD of 9 CFU/mL, and was free from interference of many ubiquitous bacteria, including *Escherichia coli*, *Bacillus subtilis*, *Staphylococcus aureus*, *Enterococcus faecalis*, and *Clostridium perfringens*. For the simultaneous detection of four types of bacteria (*Listeria monocytogenes*, *Staphylococcus aureus*, *Salmonella typhimurium*, and *P. aeruginosa*), an aptasensor exploiting Stokes and anti-Stokes photoluminescence of two QDs and two UCNPs was developed (Figure 3A) (Yüce et al., 2018). The system was composed of aptamer-modified QD and UCNP probes and partially complementary DNA-modified magnetic beads (MBs). The fluorescent signals before and after incubation with targets were detected after magnetic separation using sequential excitation at 335 nm for the QDs and 980 nm for the UCNPs. By complementing QDs with UCNPs, the problems associated with fluorescence signal overlap of QDs and the inherently poor quantum yields or limited color options of UCNPs were overcome using this multiplex sensing method.

In contrast to general fluorescent nanomaterials, polymer-based fluorescent nanomaterials display excellent biocompatibility and biodegradability, and are easy to prepare *via* self-polymerization (Zhang et al., 2015). Zhong et al. (2020) engineered a fluorescent biosensor based on dual-aptamer-labeled polydopamine–polyethyleneimine (PDA-PEI) copolymer dots (Figure 3B). The PDA-PEI copolymer dots have good aqueous dispersibility and are very stable against photobleaching, extreme pH, and high ionic strength. In the presence of *P*. *aeruginosa*, the aptamer-modified PDA-PEI dots can specifically capture the bacteria. After centrifugation, the bacterial conjugates were isolated from the solution, with the unbound aptamer-modified PDA-PEI dots remaining in the supernatant. By determining the decrease of fluorescence intensity in the supernatant, the amount of *P*. *aeruginosa* can be quantified, with the whole detection process being finished within 1.5 h. The LOD was determined to be as low as 1 CFU/mL.

These aptasensors based on fluorescent nanomaterials exhibit excellent performance in rapid detection of *P. aeruginosa* with high sensitivity and good selectivity. Although the preparation of fluorescent nanomaterials is complicated, with the further improvement and development of fabrication technologies, fluorescent nanomaterials will probably take place of organic dyes in biosensors in the future.

### Surface Plasmon Resonance-Based Aptasensor

SPR is an ultrasensitive, real-time, and label-free analytical technique (Gorodkiewicz and Lukaszewski, 2018). When light is incident at a critical angle on the interface of two media with different refractive indices, resonant absorption of free electrons in the metal occurs (Zhou et al., 2019), which leads to a diminution of the reflected light. When an analyte binds to the interface, an intensity change and/or a shift of the absorbance peak is observed, with the magnitude and position of the displacement being related to the characteristics of the surface interaction (Prabowo et al., 2018). The surface-constrained collective oscillation of free electrons produced by the interaction of light with metal nanoparticles (e.g., Au, Ag, and Cu) is known as localized surface plasmon resonance (LSPR) (Csaki et al., 2018). Compared with traditional SPR biosensors, LSPR biosensors have numerous advantages, such as low cost, miniaturization and portability, and have been widely used to detect various analytes, including small molecules, proteins and clinical biomarkers (Unser et al., 2015; Csaki et al., 2018).

**FIGURE 4:**
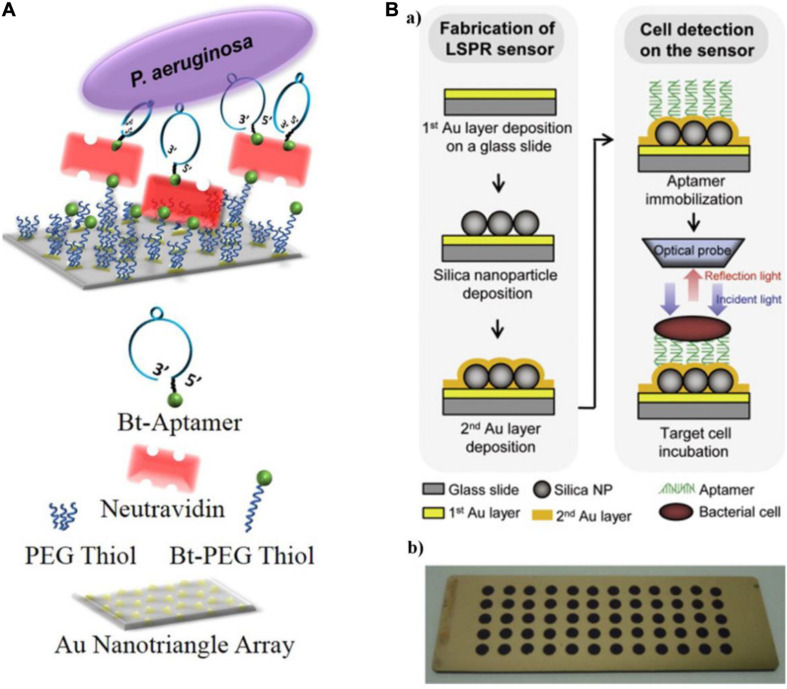
**(A)** Schematic diagram of a localized surface plasmon resonance (LSPR) sensor chip (top) and legend (bottom) (Hu et al., 2018). **(B)** Schematic representing the detection of bacterial cells using the aptamer-functionalized LSPR-based sensor **(a)**, and multispot gold-capped nanoparticle array chip **(b)**. Each spot (diameter of 3 mm) on the chip consists of Si nanoparticles (NPs) with a dielectric structure formed by the deposition of a gold layer (Yoo et al., 2015).

Hu et al. (2018) reported an aptamer-based LSPR sensing platform to detect whole-cell *P*. *aeruginosa* strain PAO1 (Figure 4A). They used nanosphere lithography to manufacture a sensor surface containing a hexagonal array of Au nanotriangles. By immobilizing aptamers on the sensor, *P. aeruginosa* cells were captured from solution onto the sensor surface, resulting in a red shift of the LSPR extinction maximum (Hu et al., 2018). The approach is extraordinarily sensitive, with a lower LOD of 10 CFU/mL. Yoo et al. (2015) used three specific aptamers as recognition elements and introduced another LSPR sensor array for the multiplexed detection of *L. acidophilus*, *S. typhimurium*, and *P. aeruginosa*. As shown in Figure 4B, a multispot gold-capped nanoparticle array chip was used in this system, which was composed of a dielectric layer comprising a thin gold (Au) layer on silica (Si) nanoparticles (NPs)-absorbed glass slide. The presence of targets manifest as a change in the LSPR peak intensity upon incubation of the chip with a sample volume of only 3 μL.

Despite the fact that LSPR-based aptasensors can accomplish label-free detection of *P. aeruginosa* with high sensitivity and good specificity, they need costly optical system as well as long preparation time.

### Surface-Enhanced Raman Scattering-Based Aptasensor

The detection of pathogens using SERS has attracted much attention, owing to its ultra-sensitive, non-destructive, fingerprint identification (Xu et al., 2019). Raman spectroscopy measures the inelastic scattering of light by analytes in the sample medium. SERS is produced by analytes adsorbed on rough metal surfaces (Zong et al., 2018).

**FIGURE 5:**
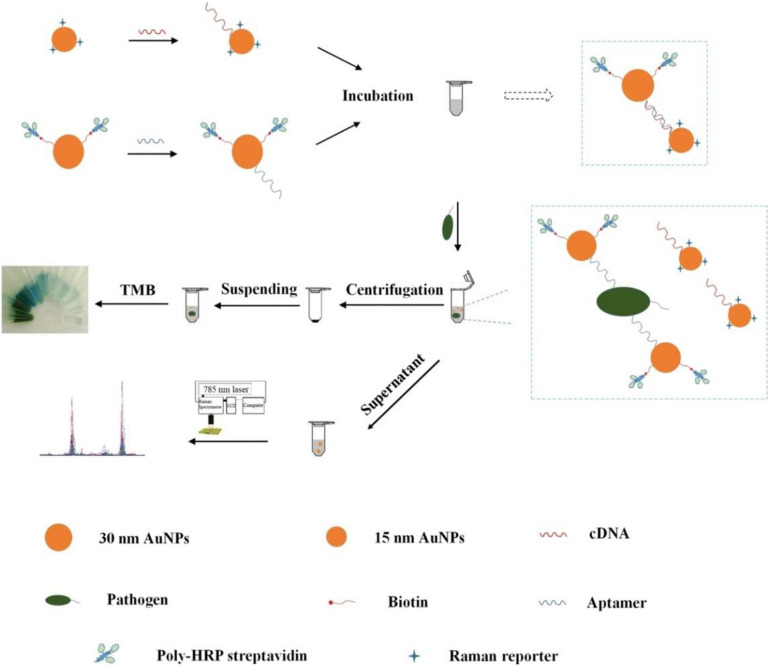
Schematic illustration of the fabrication procedure of the dual mode aptasensor for sensitive detection of *P. aeruginosa* (Wu et al., 2018).

Wu et al. (2018) fabricated a bimodal (SERS and colorimetric) aptasensor for quantitation of *P. aeruginosa* (Figure 5). A horseradish peroxidase (HRP)-linked aptamer against *P. aeruginosa* was conjugated to 30-nm gold nanoparticles (AuNPs) as a colorimetric probe, while aptamer’s cDNA fragment was coupled with 15-nm AuNPs as a SERS probe. In the absence of targets, the two probes automatically assemble into a duplex. However, upon exposure to *P. aeruginosa*, the aptamer dissociates from the cDNA and binds to its target. The previous asymmetric dimers, in which the small gaps between two differently sized AuNPs contribute to intense Raman signal enhancement, become isolated AuNPs. After centrifugation, the SERS signal from the supernatant is decreased significantly, while a colored signal is generated the from HRP-linked aptamer in the resuspended pellet after addition of 3,3′,5,5′-tetramethylbenzidine (TMB) and hydrogen peroxide. The LOD of the aptasensor is calculated to be 20 CFU/mL using SERS detection and 50 CFU/mL using colorimetric detection. The accuracy of the system is ensured by the positive colorimetric response and a concomitant negative SERS response.

In spite of the ultra-sensitivity and high accuracy of SERS-based aptasensors, the high cost of instrument and need of professional operation may restrict their common application.

## Aptasensors Based on Electrochemical Transduction

Electrochemical biosensors have a long history and remain attractive tools for identification and quantification of analytes. They demonstrate several advantages, being highly sensitive, rapid, economical, amenable to miniaturization, and easy in signal translation and operation (Maduraiveeran et al., 2018). An electrochemical biosensor is typically composed of an electrode fixed with a molecular identification element and an electrochemically active element. The presence of the target analyte will result in changes on the surface of the electrode, which are converted to electrochemical signals (current, impedance, potential, or conductance) (Li et al., 2019). Correspondingly, these biosensors can be classified into amperometric/voltammetric, impedimetric, potentiometric, and conductometric sensors (Paniel et al., 2013). The combination of these techniques with aptamers has promoted the development of aptamer-based biosensors for pathogen identification in real complex samples. Amperometry/voltammetry and impedimetry are the most frequently used electrochemical techniques for *P. aeruginosa* detection.

**FIGURE 6:**
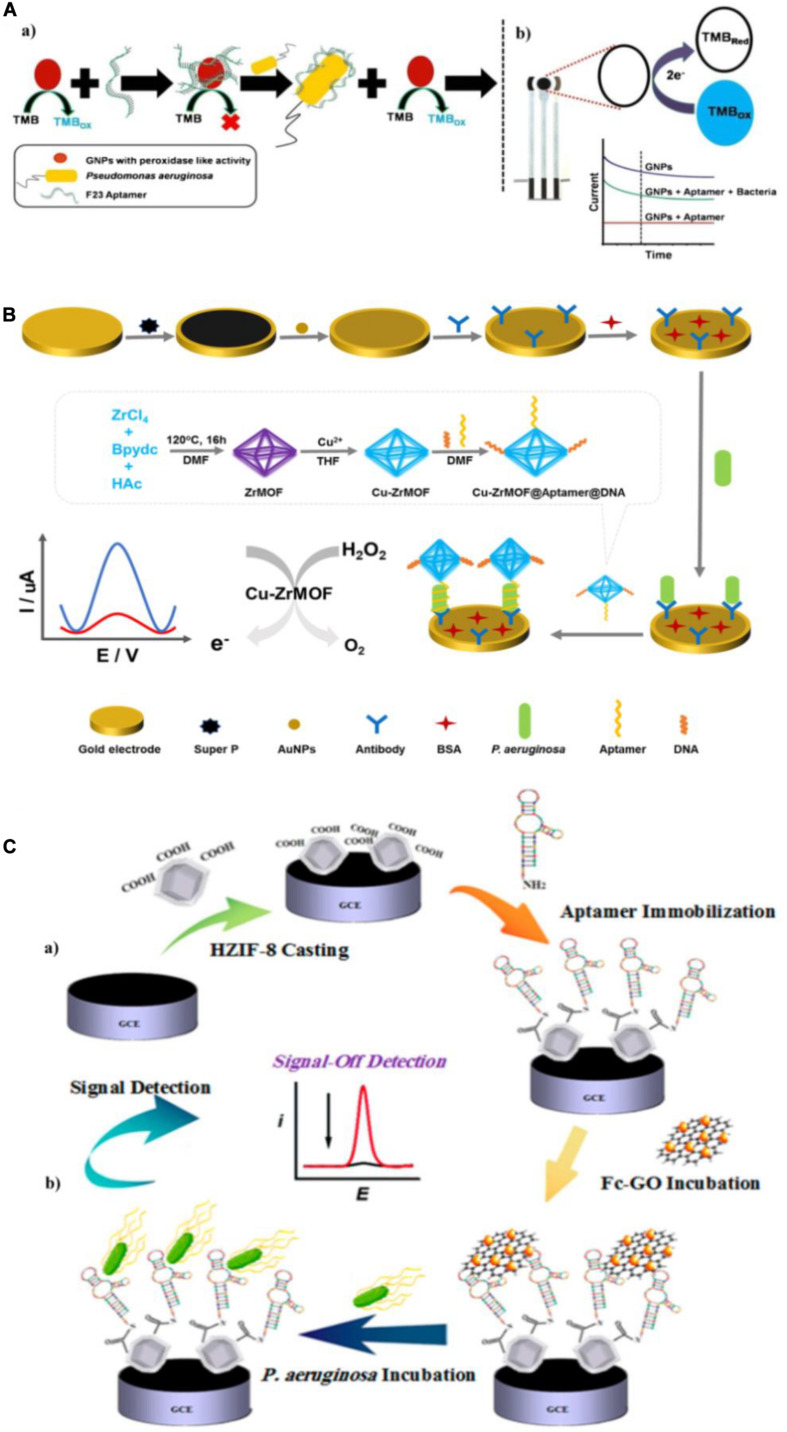
**(A)** Schematic representation of the detection of *P. aeruginosa* using the nanozyme activity of gold nanoparticles (GNPs) **(a)**, and the electrochemical detection of tetramethylbenzidine (TMB) reduction on a screen-printed carbon electrode in the presence of *P. aeruginosa* **(b)** (Das et al., 2019). **(B)** Schematic of the electrochemical detection of *P. aeruginosa* (Zhang et al., 2019b). **(C)** Schematic illustration of aptasensor fabrication **(a)** and “signal-off” detection of *P. aeruginosa* **(b)** (Shahrokhian and Ranjbar, 2019).

Amperometry/voltammetry is the most common and successful electrochemical method in research and practical applications to environment monitoring, medical analysis, and food sanitation. Several groups have constructed numerous electrochemical aptasensors of this type and used them to quantify *P. aeruginosa*. Das et al. (2019) exploited the inherent peroxidase-like nanozyme activity of gold nanoparticles (GNPs) to develop an aptamer-mediated sensor of live *P. aeruginosa* (Figure 6A). The peroxidase-mimetic activity of GNPs is inhibited by the simple adsorption of the aptamer onto their surface. However, in the presence of *P. aeruginosa*, the aptamer binds with high affinity to bacterium, restoring the peroxidase-like activity of the GNPs, which catalyzes the oxidation of TMB. With TMB as an electrochemically active material, the aptasensor was constructed using a disposable screen-printed carbon electrode (Das et al., 2019). This approach is ultrasensitive, with a lower LOD of 60 CFU/mL in water and a detection time of 10 min. Zhang et al. (2019b) constructed another novel aptasensor for rapid and sensitive detection of *P. aeruginosa*. As shown in Figure 6B, a Cu-ZrMOF (zirconium series metal-organic framework) was synthesized to provide a large surface area for aptamer/DNA adsorption and high catalytic activity. The Cu-ZrMOF/aptamer/DNA nanocomposite was used to identify *P. aeruginosa* captured on an antibody-modified electrode surface, and served as an electrochemical probe by catalyzing the decomposition of H_2_O_2_. To increase the electron transfer for satisfactory detection sensitivity, the gold electrode was coated with Super P^®^ and AuNPs. The biosensor is capable of quantifying *P. aeruginosa* with a lower LOD of 2 CFU/mL within 120 min. More importantly, this biosensor has been validated using urine samples spiked with *P. aeruginosa*, which indicates its promising potential for clinical applications. Shahrokhian and Ranjbar (2019) fabricated an electrochemical biosensor with aptamers immobilized in an engineered zeolitic imidazolate framework-8 (ZIF-8) (Figure 6C). The hollow porous ZIF-8 provided a large surface area containing abundant carboxylic acid groups in low density with a favorable open structure to immobilize a large amount of aptamers *via* amino-reactive coupling. Because of the unique π-π stacking interactions between the aptamer and GO, differential pulse voltammetric detection was used with ferrocene-graphene oxide (Fc-GO) as an electroactive indicator. Upon binding with *P*. *aeruginosa*, the aptamer undergoes conformational changes, resulting in the release of Fc-GO from the electrode surface and a change in the effective electron transfer to the electrode. Using this “signal-off” strategy, the biosensor displays a broad linear dynamic range with a lower LOD of 1 CFU/mL. The reported amperometric/voltammetric aptasensors exhibit excellent performance for quantitative determination of *P. aeruginosa* with high sensitivity, good specificity and ultralow LOD. However, the poor stability of electrode will have unfavorable effects on their application in pathogen detection.

**FIGURE 7:**
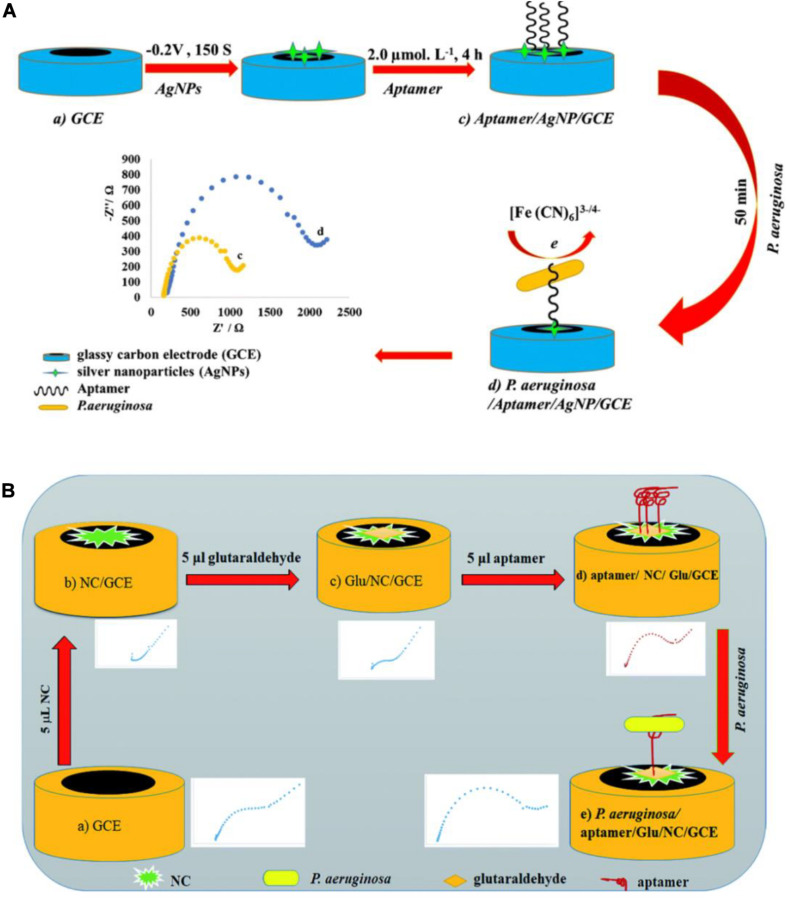
**(A)** Schematic view of the development of a *P. aeruginosa*/aptamer/silver nanoparticle/glassy carbon electrode complex for *P. aeruginosa* detection (Roushani et al., 2019). **(B)** Schematic view of the development of a *P. aeruginosa*/aptamer/glutaraldehyde (Glu)/nanosized chitosan (NC)/glassy carbon electrode complex for *P. aeruginosa* detection (Sarabaegi and Roushani, 2019).

Impedimetric biosensors have drawn much interest in recent years, owing to their reduced matrix interference, relatively simple electrical measurements, and the possibility of automation (Paniel et al., 2013). Roushani et al. (2019) constructed an impedimetric aptasensor using a glassy carbon electrode (GCE) modified with silver nanoparticles (AgNPs) (Figure 7A). Free amino groups of the aptamer were covalently coupled to the AgNP/GCE surface. The interaction between *P. aeruginosa* and aptamers significantly reduced the charge transfer from the hexacyanoferrate redox system to the surface of the electrode. The quantification of *P. aeruginosa* is carried out by monitoring the change in charge transfer resistance using the hexacyanoferrate redox system as an electrochemical probe. This assay was used to measure *P. aeruginosa* in spiked serum samples, with a lower LOD of 33 CFU/mL. Sarabaegi and Roushani (2019) designed an electrochemical aptasensor (Figure 7B) with a specific aptamer covalently linked to a GCE surface, and the hexacyanoferrate redox system as an electrochemical probe. They used nanosized chitosan (NC) particles to modify the GCE, form a sensitive layer, and improve performance. The aptasensor has a lower LOD of 3 CFU/mL and has been used to detect *P. aeruginosa* in serum samples. These impedimetric aptasensors have been recognized as promising analytical tools for *P. aeruginosa* detection. Nevertheless, they require time-consuming and costly processes to model the electrodes. More suitable materials are in demand for fabrication of impedimetric aptasensors.

The electrode material plays a key role in electrochemical biosensor construction because it directly influences the reaction speed, stability, sensitivity, electron transfer efficiency, and active site capacity. Since the 1970s, various chemical modifications to the electrode surface have been introduced to control the electrochemical process. In recent years, the use of nanotechnology to improve the sensitivity, electrochemical activity, and conductivity of electrochemical sensors has become prevalent. Compared with macroscopic materials, nanomaterials exhibit superior optical and electrical properties because of their special structures. Various nanomaterials, such as gold nanoparticles, graphene, and carbon nanotubes, have been commonly used to construct electrochemical aptasensors, owing to their large specific surface area and biocompatibility. Moreover, the emergence of new nanocomposites, such as graphene–metal nanoparticles and carbon nanotubes modified with metal nanoparticles, has advanced the development of electrochemical aptasensors. In the future, the identification of new nanomaterials and composite nanomaterials will further improve the sensitivity of electrochemical biosensors.

## Aptasensors Based on Other Signal Transduction Methods

**FIGURE 8:**
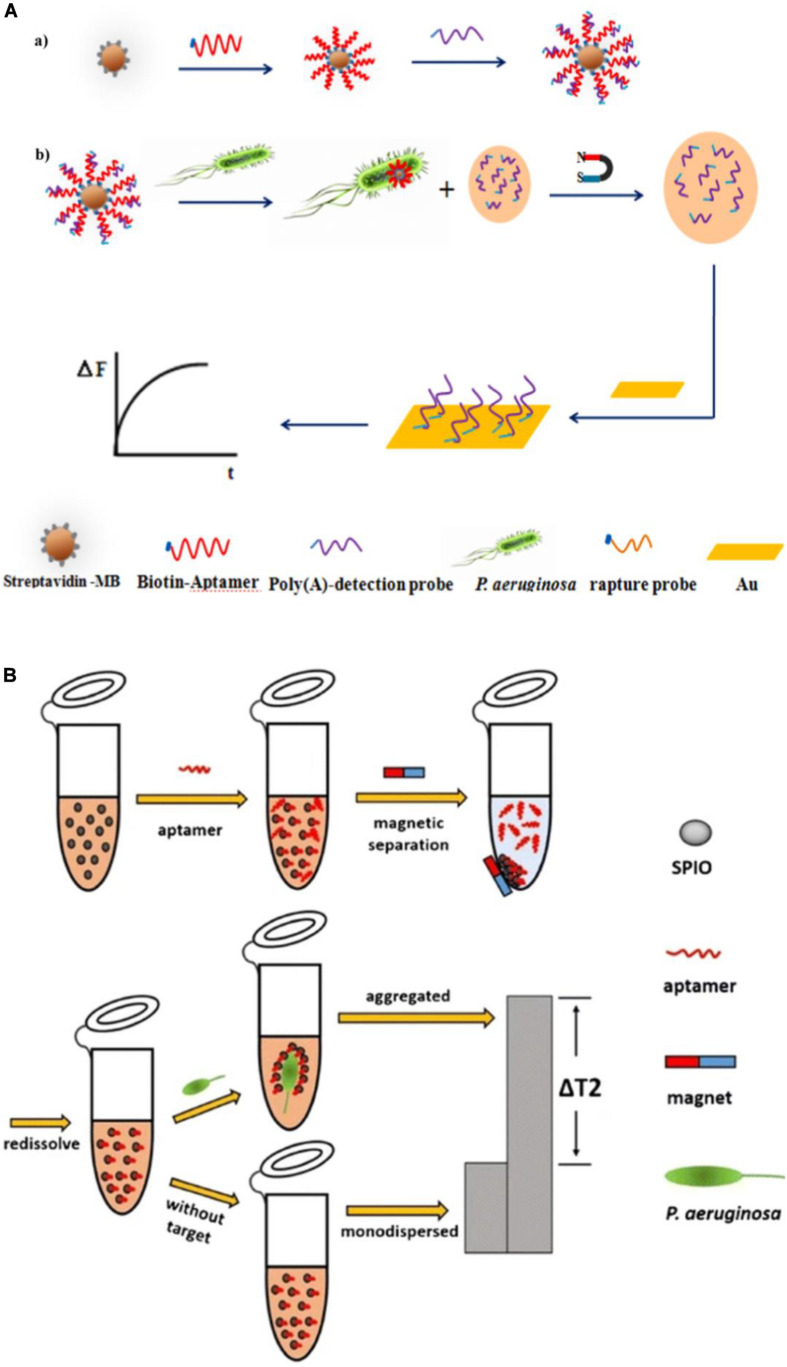
**(A)** *P. aeruginosa* detection strategy using a magnetic bead/aptamer/polyadenylated DNA complex and a gold interdigital electrode connected to a multichannel series piezoelectric quartz crystal. The preparation of magnetic bead/aptamer/polyadenylated DNA **(a)** and the detection of *P. aeruginosa* by the adsorption of released polyadenylated DNA onto the gold interdigital electrode surface **(b)** (Shi et al., 2019). **(B)** Schematic illustration of the magnetic relaxation switch aptasensor for *P. aeruginosa* detection (Jia et al., 2017).

Piezoelectric crystal biosensors, which are based on mass-sensitive transduction, can offer direct label-free analysis with excellent sensitivity and specificity. They accomplish quantification of targets by measuring changes in their oscillation frequency induced by small mass variations on surface, which can be caused by specific interactions between functional molecules, such as analyte and aptamer. Shi et al. (2019) developed an aptamer/polyadenylated DNA interdigitated gold electrode piezoelectric sensor. As shown in Figure 8A, the sandwich-type MB/aptamer/polyadenylated DNA complex was combined with a gold interdigital electrode connected to a multichannel series piezoelectric quartz crystal (Au IDE-MSPQC). In the absence of *P. aeruginosa*, polyadenylated DNA is bound to the aptamer through partially complementary interaction. In the presence of *P. aeruginosa*, the bacterium binds to the aptamer, displacing the polyadenylated DNA. After magnetic separation, the strong affinity between adenine and Au causes the adsorption of the polyadenylated DNA on the Au IDE surface, resulting in frequency shift of the MSPQC sensor. The LOD of this method is as low as 9 CFU/mL in buffer and 52 CFU/mL in simulated blood samples.

The magnetic relaxation switch (MRSw) has drawn increasing attention since 2002 (Perez et al., 2002). Its high sensitivity results from the magnetic amplification principle, and enables a wide variety of targets, including proteins, cancer cells, bacteria, and viruses to be measured (Bamrungsap et al., 2011; Ghazani et al., 2014; Chen et al., 2015; Wang et al., 2015). Aggregation of magnetic nanoparticles affects billions of surrounding water molecules, dephasing the precession of nuclear spins of water protons. The resulting change in the spin–spin relaxation time (T2) permits sensitive target detection. Jia et al. (2017) reported an MRSw-based aptasensor for *P. aeruginosa* detection (Figure 8B), with the aptamer covalently bound to superparamagnetic iron oxide (SPIO) nanoparticles. *P. aeruginosa* is captured by the aptamer, which leads to aggregation of the SPIO and a substantial increase in the T2 value of the neighboring water molecules, measured using low-field nuclear magnetic resonance. The assay has a lower LOD of 50 CFU/mL.

These aptasensors above also exhibit great potential for *P. aeruginosa* detection. They offer alternative tools for pathogen analysis in water, food, and clinical samples.

## Conclusion and Perspectives

Aptasensor is a powerful and promising sensing method for analysis of *P. aeruginosa* in food, water, and clinical samples. In this review, recent advances in the development of aptasensors for *P. aeruginosa* detection are comprehensively summarized. According to the different sensing mechanisms, representative aptasensors are classified into optical, electrochemical and other signal transduction methods, which are listed in Table 1. Optical aptasensors, including fluorescence, SPR and SERS methods, are advantageous for their properties of simplicity, rapid response and high sensitivity. Among the optical methods, the fluorescence is extensively used in quantitative determination of *P. aeruginosa*, *and* exhibits high sensitivity and good specificity. However, fluorescence are easy to be interfered by auto-fluorescence or background fluorescence. Compared with fluorescent methods, novel optical methods, such as SPR and SERS, are more stable, rapid and sensitive in detection of *P. aeruginosa*. Nevertheless, the needs of costly equipment and tedious sample preparation to some extent restrict their further application. Electrochemical aptasensors, including amperometric/voltammetric and impedimetric methods, exhibit higher sensitivity and accuracy, in comparison with optical methods. Even though they require time-consuming and costly processes to model the electrodes, electrochemical aptasensors are still recognized as the most promising analytical tools for *P. aeruginosa* detection. Apart from optical and electrochemical approaches, piezoelectric crystal aptasensors and MRSw-based aptasensors also exhibit great potential for *P. aeruginosa* quantitation.

**TABLE 1:** Comparison of analytical performance of the presented aptamer-based detection methods for *P. aeruginosa.*

Method	Material used	Linear range (CFU mL^–1^)	LOD (CFU mL^–1^)	References
Fluorescence	FITC	5.64–10^2^	5.07	Kim et al., 2013
	FAM-cDNA, MNPs	10^1^–10^8^	1	Zhong et al., 2018
	FAM-cDNA, GOQDs	1.28 × 10^3^–2 × 10^7^	100	Gao et al., 2018
	CDs, GO	10^1^–10^7^	9	Wang et al., 2018
	QDs and UCNPs, cDNA-MB	10^2^–10^6^	25	Yüce et al., 2018
	(PDA-PEI) copolymer dots	10^1^–10^7^	1	Zhong et al., 2020
LSPR	Au nanotriangle array	10^1^–10^3^	10	Hu et al., 2018
	MG-NPA	10^4^–10^9^	10^4^	Yoo et al., 2015
SERS/colorimetric	AuNPs, cDNA	10^2^–10^7^ (SERS mode) 10^2^–10^6^ (Color mode)	20 (SERS mode) 50 (Color mode)	Wu et al., 2018
Electrochemical	GNPs	6 × 10^1^–6 × 10^7^	60	Das et al., 2019
	Cu-ZrMOF/Super P, AuNPs	10^1^–10^6^	2	Zhang et al., 2019b
	HZIFs-8, GCE, Fc-GO	1.2 × 10^1^–1.2 × 10^7^	1	Shahrokhian and Ranjbar, 2019
	GCE, AgNPs	10^2^–10^7^	33	Roushani et al., 2019
	NC modified GCE	10^1^–10^7^	3	Sarabaegi and Roushani, 2019
Piezoelectric	Au IDE-MSPQC MB, polyadenylated-DNA,	8.1 × 10^1^–8.1 × 10^5^ (buffer) 1.9 × 10^2^–10^6^ (spiked sample)	9 (buffer) 52 (spiked sample)	Shi et al., 2019
MRSw	SPIO	10^2^–10^6^	50	Jia et al., 2017

*LOD, limit of detection. Ref: reference; FITC, fluorescein isothiocyanate; FAM, carboxyfluorescein; MNPs, magnetic nanoparticles; GOQDs, graphene oxide quantum dots; CDs, carbon dots; GO, graphene oxide; QDs, quantum dots; UCNPs, upconversion nanoparticles; cDNA, complementary DNA; MB, magnetic bead; PDA-PEI, polydopamine-polyethyleneimine; LSPR, localized surface plasmon resonance; SERS, surface-enhanced Raman scattering; MG-NPA, multispot gold-capped nanoparticle array; AuNPs, gold nanoparticles; GNPs, gold nanoparticles; ZrMOF, zirconium series metal-organic framework; ZIF-8, zeolitic imidazolate framework-8; GCE, glassy carbon electrode; Fc-GO, ferrocene-graphene oxide; AgNPs, silver nanoparticles; NC, nanosized chitosan particles; Au IDE-MSPQC, gold interdigital electrode connected to a multichannel series piezoelectric quartz crystal; SPIO, superparamagnetic iron oxide; MRSw, magnetic relaxation switch.*

In the past decade, the development of aptasensors is one of the most active research fields. However, detection of *P. aeruginosa* by aptasensors is still under development. Possible reasons are analyzed as follows. First, relatively few specific aptamers for *P. aeruginosa* have been obtained, which hinders the further development of aptasensors in pathogen detection. More efficient screening methods are required to identify pathogen-specific aptamers. Second, pathogens are more complicated than other targets, because multiple different components exist on their surfaces, such as proteins, polysaccharides and endotoxins. Therefore, compared with one single aptamer, a group of specific aptamers will provide better selectivity and reliability in practical aptasensor detection. Third, the stability of aptasensor requires to be further improved. Because of the flexibility in structure and charged property, aptamers are more susceptible to interferences from practical samples. Therefore, aptamers need to be further chemically optimized before aptasensor fabrication.

In the future, miniaturization, high throughput and multi-item joint testing are expected to be the focus of research. Aptasensors may someday find its place in the market to compete with the expensive antibody-based methods that are still most commonly used rapid methods at the present time.

## Author Contributions

XZ surveyed the literature and wrote the manuscript. SG conceived the topic of the manuscript and synopsis, and helped in shaping the manuscript. JW and XH provided the critical feedback and reviewed the final manuscript. All authors have read and finalized the manuscript.

## Conflict of Interest

The authors declare that the research was conducted in the absence of any commercial or financial relationships that could be construed as a potential conflict of interest.
